# A Curious Case of Blastomyces Osteomyelitis

**DOI:** 10.7759/cureus.7417

**Published:** 2020-03-25

**Authors:** Amit Sapra, Dorothy Pham, Eukesh Ranjit, Malika Q Baig, Jason Hui

**Affiliations:** 1 Family Medicine, Southern Illinois University School of Medicine, Springfield, USA

**Keywords:** osteomyelitis, knee pain, blastomycosis, fungal indection, budding yeast, atypical infection, joint swelling

## Abstract

Blastomycosis is an uncommon disease caused by the dimorphic fungus, Blastomyces dermatitidis, often found in endemic regions of Midwestern America. It can be found in forested, sandy soils, decaying vegetation, rotting wood near water sources, and even in bird feces. Most commonly, blastomycosis manifests as a pulmonary infection presenting as pneumonia, or in severe cases, respiratory distress syndrome (ARDS). Dissemination to the bone is less common but osteomyelitis of the lower thoracic and lumbar spine, ribs, skull, and long bones have been most frequently reported. Disseminated infection to the genitourinary system commonly manifests as prostatitis or epididymo-orchitis in men and as an endometrial infection or tubo-ovarian abscess in women. In the nervous system, blastomycosis can manifest as meningitis or with a cranial abscess. Having a high degree of clinical suspicion and obtaining a detailed medical and social history is important for making a diagnosis. Culturing a specimen will provide a definitive diagnosis. Sputum or tissue specimens stained in 10% potassium hydroxide under microscopy will reveal the classic appearance of B. dermatitidis (broad-based budding with a double-contoured cell wall). In mild to moderate disease without dissemination, itraconazole is the treatment of choice. In severe, life-threatening cases, patients with CNS involvement or in immunocompromised individuals, amphotericin B is the preferred initial drug of choice.

We present an interesting case of a 42-year-old African-American male with no significant past medical history who was admitted initially for suspicion of cellulitis/septic arthritis and was started on broad-spectrum antibiotics. However, he was eventually found to have Blastomyces osteomyelitis.

## Introduction

Blastomycosis is a disease that is caused by a dimorphic fungus, Blastomyces dermatitidis, and is often misdiagnosed [1-3]. Blastomycosis is correctly suspected at the first clinical evaluation in only a small percentage of patients as it can mimic a variety of commonly seen conditions. Pulmonary blastomycosis is diverse and can present in multiple forms. It can resemble bacterial community-acquired pneumonia, tuberculosis, lung cancer, and acute respiratory distress syndrome (ARDS) [2].

Extrapulmonary infection with Blastomyces dermatitidis is diverse and has many different manifestations. Most commonly, skin or subcutaneous lesions are found with either a warty or in an ulcerative form. Cases have been misidentified as squamous cell carcinoma, keratoacanthoma, pyoderma gangrenosum, or as panniculitis [4]. The bone is the second most common site of dissemination. Osteomyelitis of the lower thoracic and lumbar spine, ribs, skull, and long bones have been most frequently reported with blastomycosis, although essentially any bone can be affected. The infection has the potential to spread to adjacent joints, resulting in septic arthritis subsequently [2].

The diagnosis of blastomycosis requires a high degree of clinical suspicion, and a detailed history of occupational or recreational exposure in endemic areas can aid in an early diagnosis. Blastomyces dermatitidis causes clinical manifestations that mimic many other commonly seen conditions, and that is why blastomycosis has also been referred to as 'The Great Pretender' [5].

We present the case of a 42-year-old African-American male with no significant past medical history who was admitted for increasing right knee pain for three weeks. Although his presentation was consistent with cellulitis/septic arthritis, he was diagnosed with osteomyelitis secondary to blastomycosis, which we had least expected.

## Case presentation

Our patient is a 42-year-old African-American male with no significant past medical history who was admitted for increasing right knee pain over a period of three weeks. The patient had no history of trauma to the area but reported hitting it with a "pan" at work, as he works as a chef. The pain was not radiating and was moderate to severe in intensity. He had been taking nonsteroidal anti-inflammatory drugs (NSAIDs) for pain with minimal improvement and could not bear weight on the right knee due to pain. 

The patient denied any history of sexually transmitted diseases (STDs), recent infection, travel, or sick contacts. The patient has lived in Illinois his entire life and has not seen a doctor in over 20 years. His social history was significant for smoking half a pack of cigarettes daily, occasional marijuana use, and alcohol use in the past. There was no history of intravenous (IV) drug use. 

On physical examination, the patient's vital signs were stable. His right knee was swollen and tender to the touch with swelling in the medial aspect of the proximal tibia. There were no cuts, abrasions, or signs of obvious trauma locally. The knee had a limited range of motion with pain on movement. Ligament laxity was not assessed because of the discomfort the patient was in.

His labs showed elevated inflammatory markers. His C-reactive protein was 205.8 (normal: < 3 mg/L) and the erythrocyte sedimentation rate was 101 (range: 1 to 13 mm/hr). His white blood cell count was within normal limits.

**Figure 1:**
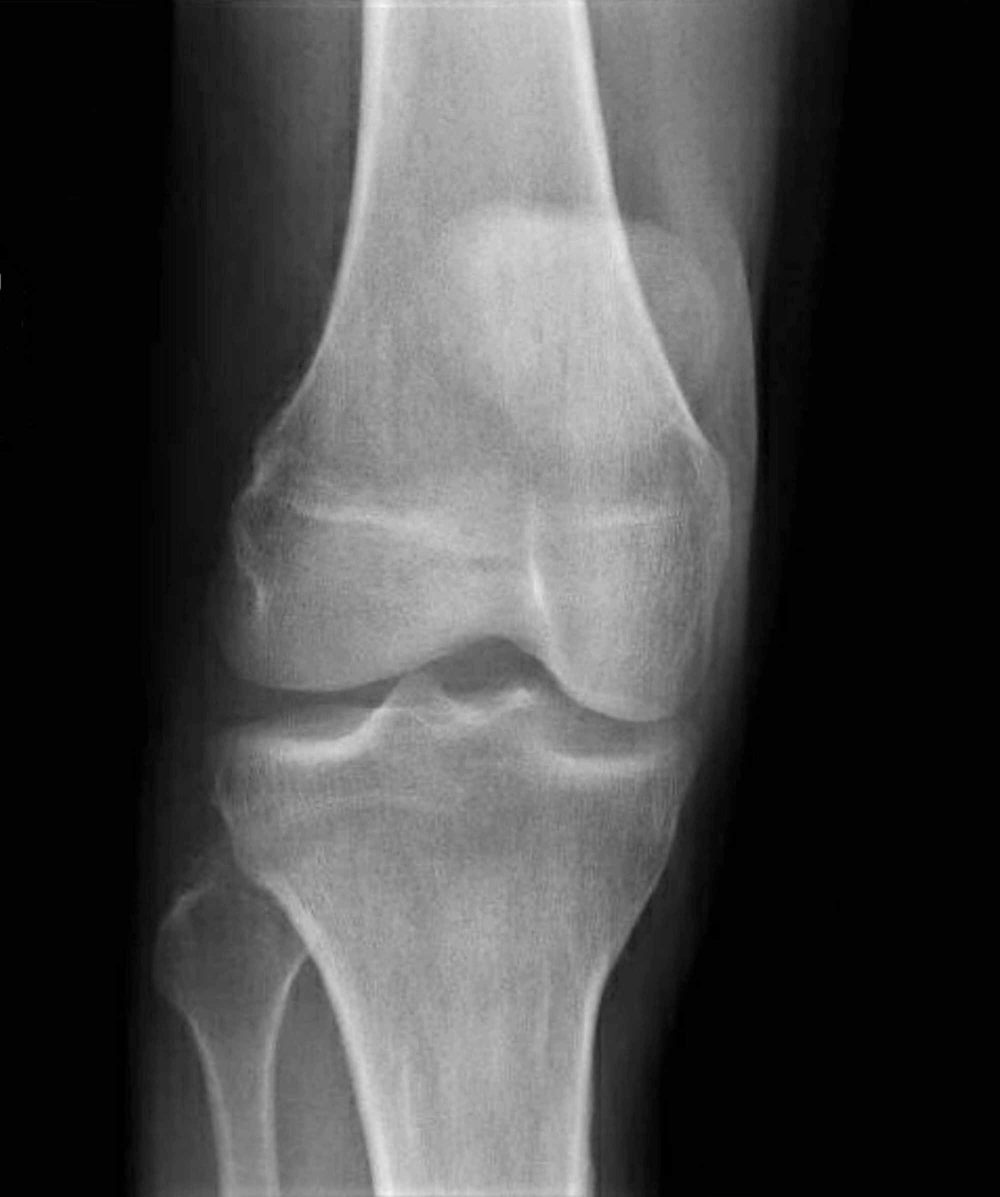
Anteroposterior (AP) view of the right knee was reported as being normal

X-ray of the right knee showed no bony abnormality (Figure 1).

**Figure 2:**
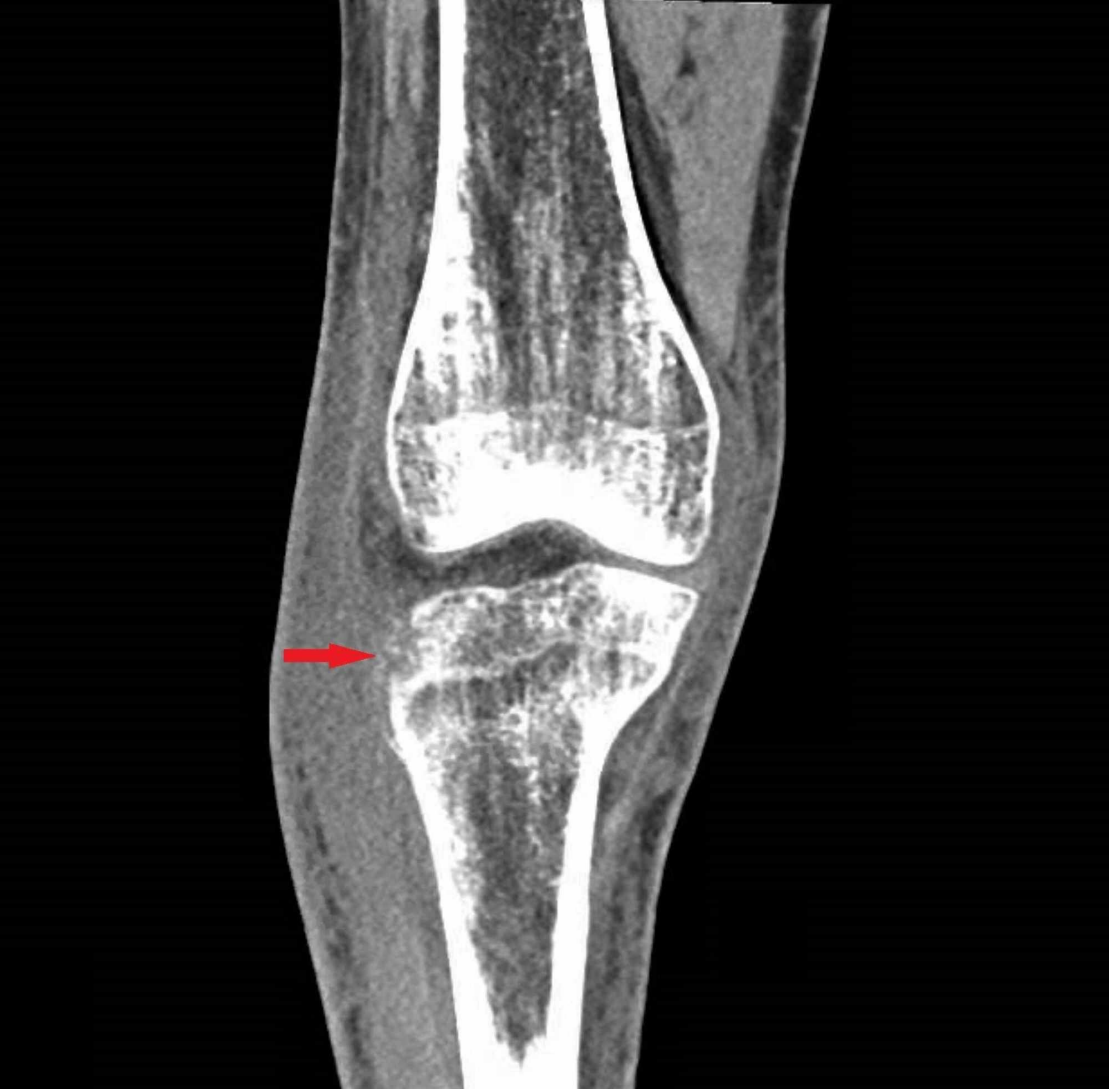
Computed tomography (CT) scan of the right lower extremity showing cortical irregularity (red arrow) around the proximal tibial metaphysis

Computed tomography (CT) of the right knee demonstrated two positive findings: 1) prominent soft tissue swelling adjacent to the lateral aspect of the proximal tibial metaphysis with some associated underlying cortical irregularity of the tibia at this location. The findings were suspicious for a soft tissue infection with resultant secondary osteomyelitis of the proximal tibia; 2) small knee joint effusion (Figure 2).

**Figure 3:**
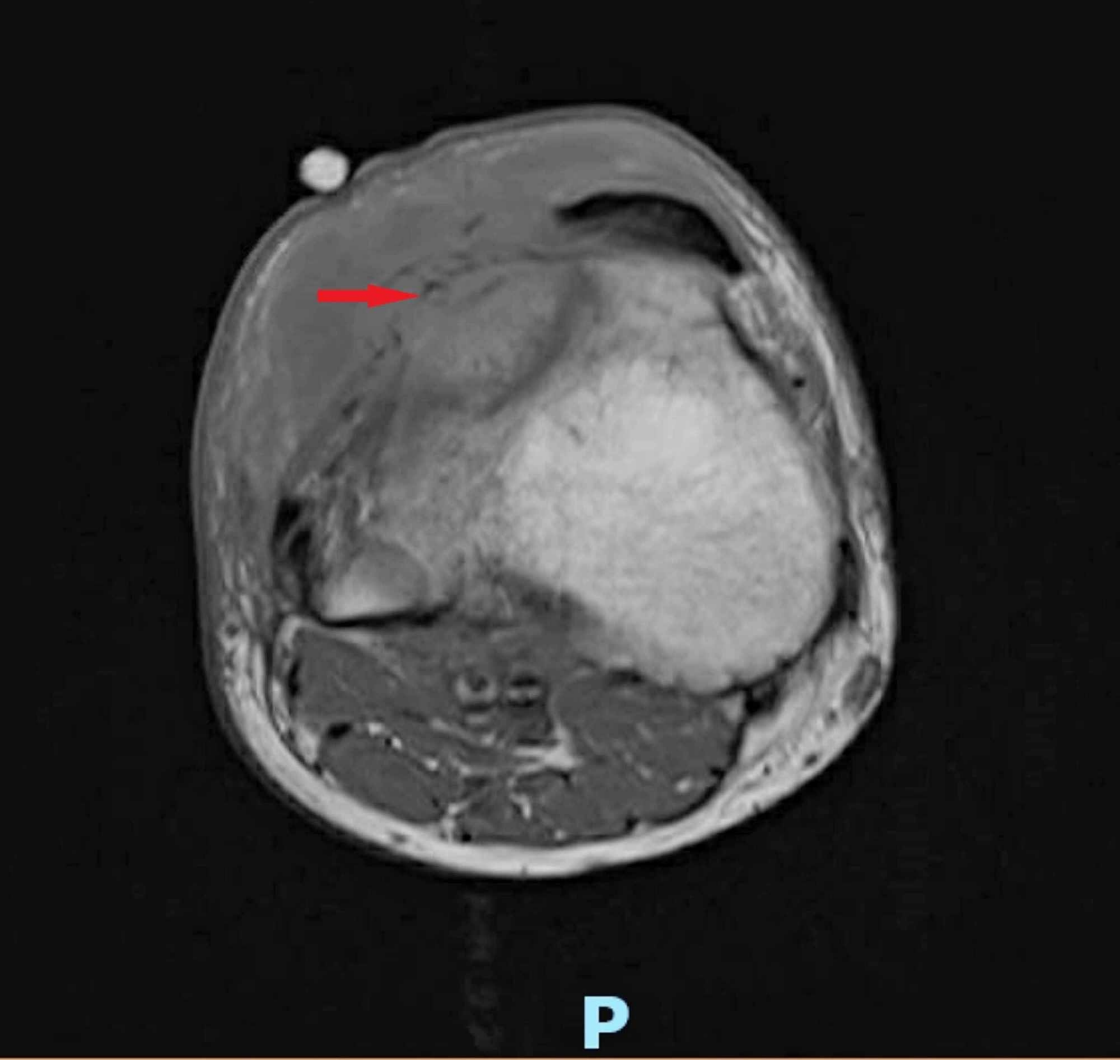
Magnetic resonance imaging (MRI) scan of the right knee shows osteomyelitis involving the lateral tibial plateau, extending inferiorly into the tibial tubercle, along with a 6 x 2 cm abscess within the subcutaneous tissues anterolaterally at the level of the proximal tibia (red arrow)

Magnetic resonance imaging (MRI) of the right knee demonstrated three positive findings: 1) osteomyelitis involving the lateral tibial plateau extending inferiorly into the tibial tubercle. A portion of the lateral tibial plateau appeared to be necrotic; 2) small to moderate knee joint effusion; 3) a 6 x 2 cm abscess as seen within the subcutaneous tissues anterolaterally at the level of the proximal tibia (Figure 3).

**Figure 4:**
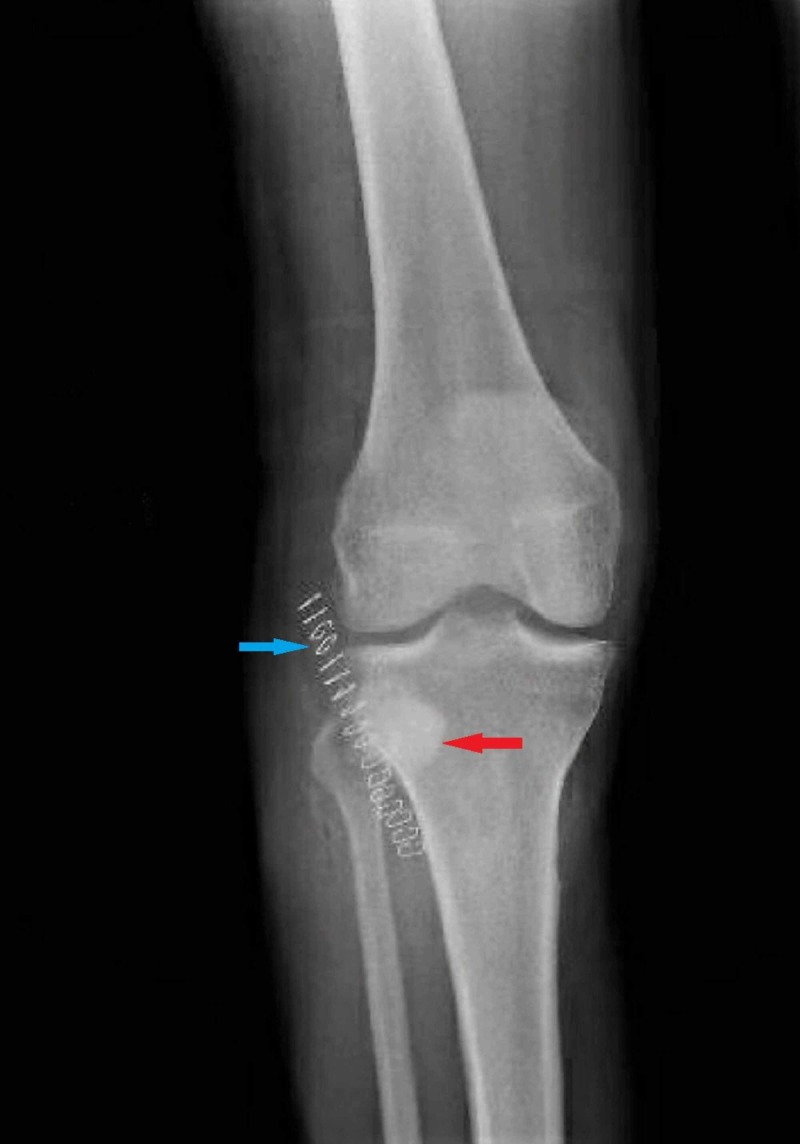
Postoperative x-ray (anteroposterior (AP) view) of the knee showing radio-opaque antibiotic cement (red arrow) in the lateral tibial condyle and staples (blue arrow)

Blood cultures were drawn, and the patient was started on IV daptomycin, cefepime, and metronidazole. Orthopedic surgery was consulted, and the patient underwent arthroscopic incision and drainage with debridement of the right proximal anterolateral tibia for the abscess with associated osteomyelitis. Antibiotic cement was placed in the abscess cavity, and samples were sent for bacterial, acid-fast bacilli (AFB), and fungal cultures. The patient reported a significant improvement in the pain and swelling of the joint following the procedure (Figure 4).

**Figure 5:**
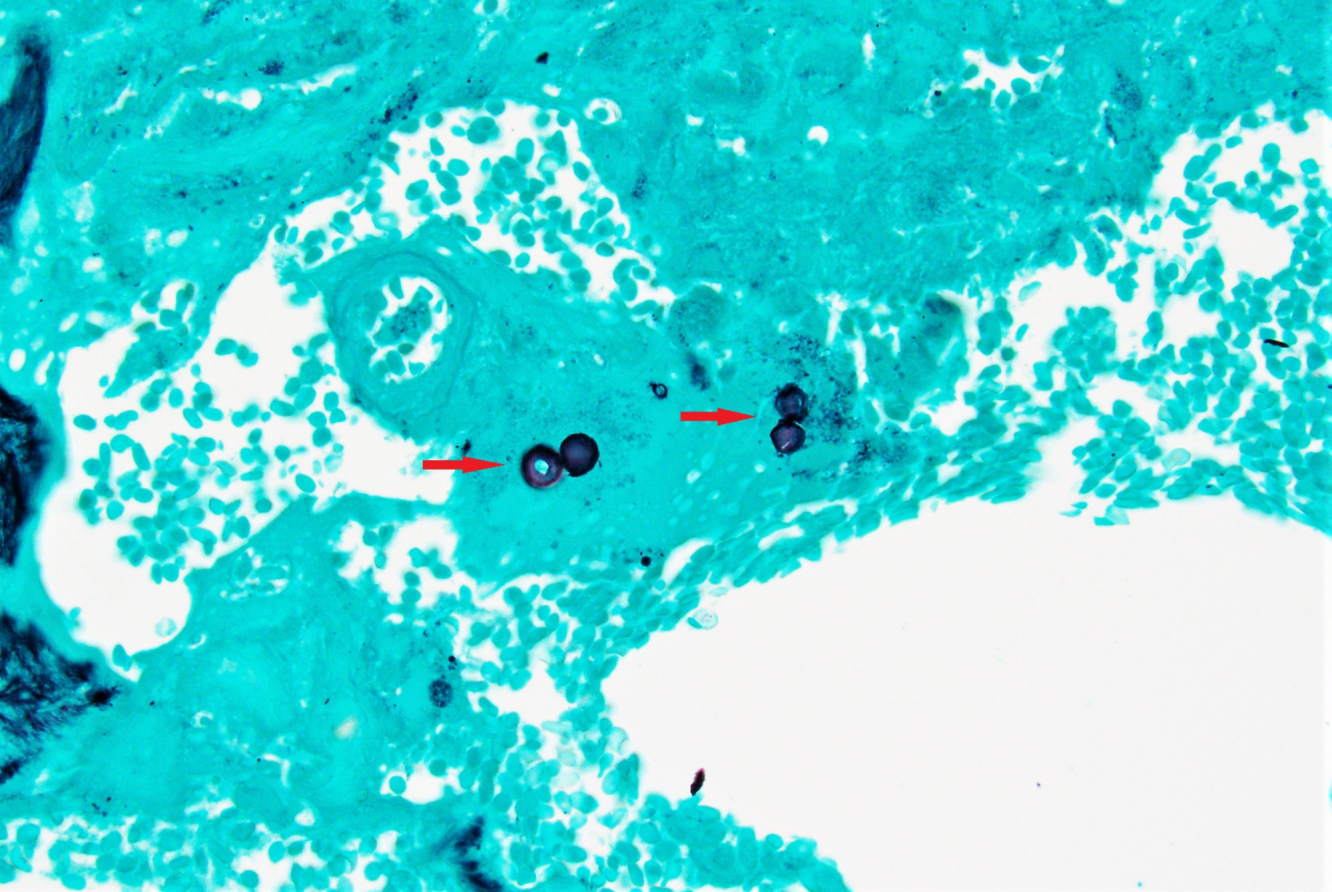
Grocott's methenamine silver (GMS) stain of the bone biopsy sample showing broad-based budding yeast suggestive of Blastomyces species (red arrow)

Intraoperative fungus culture grew broad-based budding yeasts suggestive of the Blastomyces species. The histopathology report was consistent with acute and chronic granulomatous osteomyelitis, and Grocott's methenamine silver (GMS) stain showed Blastomyces. Our patient was determined to have a right proximal tibial abscess and osteomyelitis secondary to blastomycosis (Figure 5).

The Department of Infectious Diseases was consulted, and they assessed the patient was likely to have disseminated blastomycosis due to pulmonary and bone involvement, as his CT scan of the chest showed hilar lymphadenopathy. He was started on IV amphotericin B for disseminated blastomycosis for a total of four weeks to clear the infection with close monitoring of the potassium and magnesium levels since amphotericin B is known to cause hypokalemia and hypomagnesemia. The broad-spectrum antibiotics that had been started initially were continued. The patient was determined to be an immunocompetent host as human immunodeficiency virus (HIV) screen and viral load were negative, and the immunoglobulin and T-lymphocyte counts were within normal limits. Bacterial cultures remained negative, and all antibiotics were subsequently discontinued. 

The patient underwent daily dressing changes, as per the orthopedic team, and had reaccumulation of the abscess twice, requiring drainage each time. He was discharged on IV amphotericin B for a total of four weeks and was counseled on smoking cessation. All other cultures and infectious workup, including HIV and hepatitis C, remained negative. The patient was able to ambulate and physical therapy was ordered on an ongoing basis upon discharge.

After discharge, the patient continued to recover well, receiving daily IV amphotericin B infusions. His treatment was complicated by low potassium and magnesium levels, getting as low as 2.1 mmol/L (normal: 3.5 - 5.0 mmol/L) and 1.2 mmol/L (normal: 0.62 - 0.99 mmol/L), respectively, and the patient was started on daily supplementation. 

The patient was advised to complete a four-week course of IV amphotericin B and then start on a six-month regimen of oral itraconazole. The patient has been showing a steady recovery since the antifungal treatment was started and has a close follow-up planned with his orthopedic surgeon, infectious disease specialist, and primary care physician.

## Discussion

Epidemiology

Blastomycosis is an uncommon fungal infection caused by a dimorphic fungus, Blastomyces dermatitidis, that is endemic in the Ohio and Mississippi River Valleys, Great Lakes region, and the southeastern United States, as well as in the Canadian provinces of Manitoba and Ontario [1-3]. Blastomycosis has an annual incidence of approximately 0.2 - 1.94 cases per 100,000 persons in North America, although this could be underestimated as a result of underreporting due to under-diagnosis of the disease [2]. According to the Center for Disease Control, states that are required to report blastomycosis cases are the following: Arkansas, Louisiana, Michigan, Minnesota, and Wisconsin [6].

Blastomycosis is found in forested, sandy soils, decaying vegetation, rotting wood near water sources, and even in bird feces. Occupations that disrupt soil, like construction or forestry, and outdoor recreational activities, like compost piling, hunting, canoeing, boating, tubing, and fishing, have all been associated with the disease [2]. Blastomycosis typically affects individuals between the ages of 25 and 50, and there is a slightly higher prevalence in men than in women [4]. 

Similar to histoplasmosis, coccidioidomycosis, and cryptococcosis, blastomycosis can occur among immunocompromised individuals with a history of organ transplantation, immunotherapy (tumor necrosis factor (TNF)-alpha inhibitors, malignancy, HIV/acquired immune deficiency syndrome (AIDS)), or those who are in a partial immunosuppressive state, such as pregnant women [2, 7]. Blastomycosis can also be found among healthy individuals. Most individuals who have blastomycosis are immunocompetent; however, those who are immunocompromised have a more severe, life-threatening course of the disease [2].

Clinical presentation

Only 50% of infected individuals have subclinical or asymptomatic illness [1]. In 80% of symptomatic patients, blastomycosis manifests as a pulmonary infection following inhalation of spores, presenting most commonly as pneumonia, or in severe cases, as respiratory distress syndrome (ARDS) [2]. Twenty-five to 40% of patients have extrapulmonary symptoms with dissemination by hematogenous or lymphatic spread to the following organ systems (listed in order from the most common to the least common): the skin (reported in 40% - 80% of patients), bones (reported in approximately 25% of patients), the genitourinary tract (reported in less than 10% of patients), and the central nervous system (reported in less than 5% - 10% of immunocompromised patients) [2]. 

The bone is the second most common site of dissemination. Osteomyelitis of the lower thoracic and lumbar spine, ribs, skull, and long bones has been most frequently reported with blastomycosis, although essentially any bone can be affected. The infection has the potential to subsequently spread to adjacent joints, resulting in septic arthritis [2]. Disseminated infection to the genitourinary system commonly manifests as prostatitis or epididymo-orchitis in men and as endometrial infection or tubo-ovarian abscess in women (via transmission from a man with a blastomycosis skin lesion on the penis) [4, 8]. In the nervous system, blastomycosis can manifest as meningitis or with a cranial abscess. Essentially, blastomycosis can disseminate from the lungs to any organ system [8]. 

Differential diagnoses

Acute and chronic blastomycosis can mimic other diagnoses in terms of symptoms and appearance on imaging. In acute pulmonary blastomycosis, consolidation is the most common chest radiographic finding, and therefore, can be misdiagnosed as bacterial community-acquired pneumonia (CAP). Cough can be either productive or non-productive with associated symptoms of fever, chills, headache, dyspnea, chest pain, and malaise. When left undiagnosed and untreated, chronic blastomycosis can develop and mimic a lung neoplasm or tuberculosis [2].

In one reported case, a patient with an abnormal mammogram and computerized CT was initially mistaken to have breast carcinoma with metastasis to the spine [4]. In another case, a patient with cutaneous symptoms (but without pulmonary symptoms) was misdiagnosed with having squamous cell carcinoma [9].

In more than 40% of patients, it took more than one month after the initial presentation until the correct diagnosis of blastomycosis was made [2].

Diagnosis

Having a high degree of clinical suspicion and obtaining a detailed medical and social history is important for making a diagnosis. History may include a place of residence near freshwater or waterways, travel, outdoor activities (fishing, canoeing, rafting), hobbies, road construction, community compost piling, and household pets [2]. Blastomycosis is not considered contagious among humans, as it generally is not transmitted from person to person [3].

A culture of a specimen will provide a definitive diagnosis. A culture of bronchoalveolar lavage or sputum has a 92% and 86% diagnostic yield, respectively [2]. B. dermatitidis grows slowly, however, and it can take approximately five to 14 days to be visualized. Sputum or tissue specimens stained in 10% potassium hydroxide under microscopy will reveal the classic appearance of B. dermatitidis: broad-based budding with a double-contoured cell wall [2]. Antibody tests, antigen detection (urine, cerebrospinal fluid (CSF), bronchoalveolar lavage), nucleic acid amplification tests, and skin tests have not been proven to be useful in making a diagnosis [8].

Management

In mild to moderate disease without dissemination, itraconazole is the treatment of choice due to it having less adverse side effects [4]. A typical regimen is itraconazole, 600 mg given orally daily for three days, followed by 200 to 400 mg daily for six to 12 months) [2]. In severe, life-threatening cases, patients with central nervous system (CNS) involvement or immunocompromised individuals, amphotericin B is the preferred initial drug of choice. Amphotericin B is used at a high dose of 0.7 to 1 mg/kg/day IV infusion for approximately one to two weeks. If there are signs of improvement, therapy can be switched to itraconazole for a total duration of antifungal therapy of at least 12 months [2, 8].

Prognosis

Successful recovery from blastomycosis, without relapse or mortality, occurs in 80% - 95% of cases [7]. Studies have shown a 95% cure rate in those completing therapy with itraconazole for mild to moderate disease, and a 91% cure rate with amphotericin B for severe disease with a low relapse rate when given at a dose of at least 1.5 gm [8]. 

Also, older individuals have more reported deaths due to blastomycosis than younger individuals. The mean age at death was 60.8 years [10]. Early diagnosis and prompt treatment of blastomycosis can improve the prognosis but that is more likely to occur in highly endemic areas, such as those in Wisconsin, where clinicians are more likely to encounter blastomycosis, and therefore, have a higher degree of clinical suspicion [11]. 

Prevention

There are currently no existing recommendations on lifestyle or behavioral changes that would reduce the incidence of blastomycosis. There is also no available vaccine to prevent blastomycosis [8]. 

## Conclusions

Our case report demonstrates just one of the many different ways that blastomycosis can present. There are no findings on imaging or examination that would be pathognomonic for blastomycosis, thereby highlighting the importance of having a high degree of clinical suspicion and to obtain a detailed medical and social history to form a diagnosis. The condition is often misdiagnosed as it can mimic a variety of commonly seen conditions. Early diagnosis and subsequent prompt treatment of blastomycosis can improve the prognosis. Search for the causative organism by microscopy (using special stains when indicated) and culture should be considered, even in the patients who present with a classic picture of other commonly seen illnesses.

## References

[1] Miceli A, Krishnamurthy K (2020). Blastomycosis. StatPearls [Internet].

[2] McBride JA, Gauthier GM, Klein BS (2017). Clinical manifestations and treatment of Blastomycosis. Clin Chest Med.

[3] Stavrakis C, Narayan A, Voronel O (2015). Cerebral blastomycosis: radiologic-pathologic correlation of solitary CNS blastomycosis mass-like infection. J Clin Imaging Sci.

[4] Bradsher RW Jr (2014). The endemic mimic: blastomycosis an illness often misdiagnosed. Trans Am Clin Climatol Assoc.

[5] Lemos LB, Baliga M, Guo M (2002). Blastomycosis: the great pretender can also be an opportunist. Initial clinical diagnosis and underlying diseases in 123 patients.. Ann Diagn Pathol.

[6] (2020). Fungal Diseases: Blastomycosis Statistics. http://www.cdc.gov/fungal/diseases/blastomycosis/statistics.html.

[7] Surprenant D, Kaniszewska M, Hutchens K, Go C, O'Keefe P, Swan J, Tung R (2015). Blastomycosis and pregnancy: an unusual postpartum disease course. Case Rep Dermatol.

[8] Saccente M, Woods GL (2010). Clinical and laboratory update on blastomycosis. Clin Microbiol Rev.

[9] Kruse A, Zwahlen RA, Bredell MG, Gengler C, Dannemann C, Grätz KW (2010). Primary blastomycosis of oral cavity. J Craniofac Surg.

[10] Khuu D, Shafir S, Bristow B, Sorvillo F (2014). Blastomycosis mortality rates, United States, 1990-2010. Emerg Infect Dis.

[11] Baumgardner DJ, Temte JL, Gutowski E, Agger WA, Bailey H, Burmester JK, Banerjee I (2011). The differential diagnosis of pulmonary blastomycosis using case vignettes: a Wisconsin Network for Health Research (WiNHR) study. WMJ.

